# Performance and outcomes of transvenous rotational lead extraction: Results from a prospective, monitored, international clinical study

**DOI:** 10.1016/j.hroo.2021.02.005

**Published:** 2021-03-02

**Authors:** Saumya Sharma, Byron K. Lee, Anuj Garg, Robert Peyton, Brian T. Schuler, Pamela Mason, Peter Paul Delnoy, Mark M. Gallagher, Ramesh Hariharan, Raymond Schaerf, Ruirui Du, Nina D. Serratore, Christoph T. Starck

**Affiliations:** ∗ Department of Cardiac Electrophysiology, The University of Texas Health Science Center at Houston, Houston, Texas; † Division of Cardiology, Electrophysiology, and Arrhythmia Service, University of California, San Francisco, San Francisco, California; ‡ Heart and Vascular Institute, Carle Foundation Hospital, Urbana, Illinois; § Carle Illinois College of Medicine, University of Illinois, Urbana Champaign, Champaign, Illinois; ‖ UNC REX Healthcare, Cardiac Surgical Specialists, Raleigh, North Carolina; ¶ Electrophysiology, Wellspan Medical Group, York, Pennsylvania; # Division of Cardiovascular Medicine, University of Virginia Health System, Charlottesville, Virginia; ∗∗ Department of Cardiology, Isala Hospital, Zwolle, The Netherlands; †† Cardiology Clinical Academic Group, St. George’s University Hospitals NHS Foundation Trust, London, United Kingdom; ‡‡ Providence Saint Joseph Medical Center, Burbank, California; §§ Cook Research Incorporated, West Lafayette, Indiana; ‖‖ Department of Cardiothoracic and Vascular Surgery, German Heart Center Berlin, Berlin, Germany

**Keywords:** Complication, Clinical success, Mechanical transvenous lead extraction, Prospective clinical trial, Rotational extraction sheath

## Abstract

**Background:**

Transvenous lead extraction (TLE) plays a critical role in managing patients with cardiovascular implantable electronic devices. Mechanical TLE tools, including rotational sheaths, are used to overcome fibrosis and calcification surrounding leads. Prospective clinical data are limited regarding the safety and effectiveness of use of mechanical TLE devices, especially rotational tools.

**Objective:**

To prospectively investigate the safety and effectiveness of mechanical TLE in real-world usage.

**Methods:**

Patients were enrolled at 10 sites in the United States and Europe to evaluate the use of mechanical TLE devices. Clinical success, complete procedural success, and complications were evaluated through follow-up (median, 29 days). Patient data were source verified and complications were adjudicated by an independent clinical events committee (CEC).

**Results:**

Between October 2018 and January 2020, mechanical TLE tools, including rotational sheaths, were used to extract 460 leads with a median indwell time of 7.4 years from 230 patients (mean age 64.3 ± 14.4 years). Noninfectious indications for TLE were more common than infectious indications (61.5% vs 38.5%, respectively). The extracted leads included 305 pacemaker leads (66.3%) and 155 implantable cardioverter-defibrillator leads (33.7%), including 85 leads with passive fixation (18.5%). A bidirectional rotational sheath was needed for 368 leads (88.0%). Clinical success was obtained in 98.7% of procedures; complete procedural success was achieved for 96.3% of leads. CEC-adjudicated device-related major complications occurred in 6 of 230 (2.6%) procedures. No isolated superior vena cava injury or procedural death occurred.

**Conclusion:**

This prospective clinical study demonstrates that use of mechanical TLE tools, especially bidirectional rotational sheaths, are effective and safe.

## Graphical abstract

**Figure:**
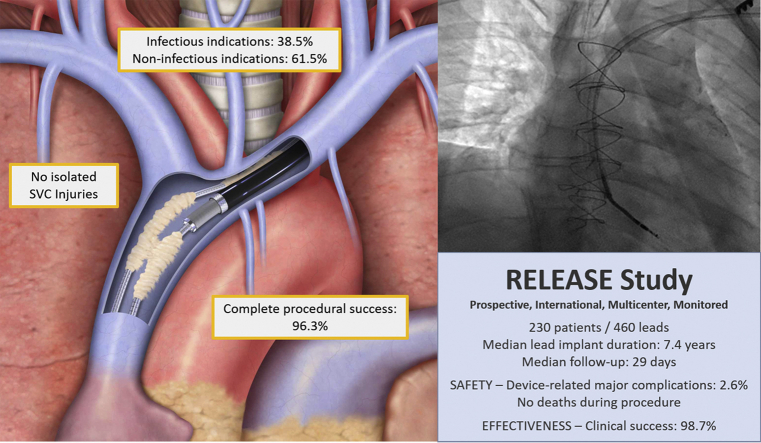

**Key Findings**

▪ The use of mechanical lead extraction devices, especially bidirectional rotational sheaths, is effective, efficient, and safe.
▪ This study represents an advance in transvenous lead extraction (TLE) trial design, as this study emphasizes the need for comprehensive follow-up and adjudication of all complications by an independent clinical events committee with experience in TLE procedures.
▪ The prospective, multicenter, and fully monitored study design provided a more accurate understanding of the effectiveness and safety of lead extraction procedures.

## Introduction

Over the past decade, implantation of cardiovascular implantable electronic devices has increased. As a natural result of this increase, there has been a rise in the number of transvenous lead extraction (TLE) procedures.^1^ Fibrotic adhesions develop on chronically implanted leads, which increase the complexity of TLE. Despite improvements in technologies and techniques, TLE remains a challenging procedure with a risk of potentially life-threatening complications.^1^^,^^2^

Historically, lead extraction has been considered a complex open surgical procedure performed as a last resort and associated with significant mortality. The development of “locking stylets” and laser cutting sheaths provided new strategies for TLE. Laser-assisted TLE devices were first introduced in the mid-1990s. Laser TLE tools have been used extensively, with several studies supporting the success of laser TLE.^3^^,^^4^ In 2006, a mechanical rotational lead extraction technique utilizing the mechanical dilator sheath (Evolution®; Cook Medical, Bloomington, IN) was introduced. This mechanical technique was developed to overcome the fibrosis and calcifications observed in patients with chronically implanted leads. More recently, the bidirectional rotational TLE sheath (Evolution RL; Cook Medical, Bloomington, IN) was introduced with a protective outer sheath, providing enhanced tactile feedback, power control, and sheath flexibility. Considering these benefits, mechanical rotational TLE emerged as an effective alternative to laser techniques.

In 2010, Hussein and colleagues^5^ reported their initial experience with the Evolution Mechanical Dilator Sheath. Subsequently, multiple studies showed that mechanical rotational TLE devices are efficient,^6^^,^^7^ have high success rates,^6^^,^^8^^,^^9^ and are safe^6^^,^^8^, ^9^, ^10^ for extracting chronically implanted leads. Mechanical rotational sheaths were also associated with reduced mortality risk compared to laser sheaths.^11^ Moreover, comparable clinical and complete procedural success rates were observed with laser and mechanical rotational TLE approaches.^12^

In recent years, the safety and effectiveness of mechanical TLE, including the use of rotational TLE tools, has been established in large-scale, retrospective single-center^13^ and multicenter experiences^8^^,^^14^; however, a prospective, international clinical study to evaluate both procedural outcomes and safety during patient follow-up was warranted.

## Methods

This prospective, postmarket clinical study included patients treated with Cook® mechanical TLE devices (Cook Medical, Bloomington, IN) at 10 medical centers in the United States and Europe. Nine of these centers were considered high-volume (30 or more TLE procedures per year) and 1 was considered low-volume (fewer than 30 TLE procedures per year) lead extraction facilities.^1^^,^^15^ Each center was limited to a maximum of 20% of total enrollment to minimize bias that may be introduced if the majority of patients are enrolled at 1 or 2 centers. This study was conducted in accordance with the Declaration of Helsinki, ISO 14155, and applicable local regulations. All local institutional review boards (US) / ethics committees (EU) approved the study protocol and all patients provided written informed consent prior to enrollment. The Transvenous Lead Removal Using the Cook Evolution® LEAd Extraction SystEm (RELEASE) Post-Market Clinical Study was sponsored by Cook Medical and is registered at https://www.clinicaltrials.gov with the identifier NCT03688412.

### Patients and data acquisition

Patients who required a TLE procedure were eligible for enrollment if the physician intended to use Cook Medical mechanical TLE devices. For patients enrolled in the RELEASE study, at least 1 lead per patient was required to be removed using a Cook mechanical TLE device. Cook mechanical TLE devices were used in all lead extraction procedures except for those leads requiring only simple tension for removal. Eligibility was limited to adult patients enrolled in the study (at least 18 years of age), with at least 1 lead having an indwell time greater than 1 year, and who provided written informed consent. Prospective data collection included preprocedure, procedure, and follow-up patient parameters documented in electronic case report forms (CRFs). Data were recorded during each TLE procedure by trained research staff including, but not limited to, the research coordinator. The exact start and stop time per lead extraction, as well as device use, were documented in the CRF in real time. Follow-up assessments were performed according to the standard of care per medical center at approximately 4 weeks after the study procedure, either in person or via telephone. Study data were source verified by an independent clinical monitoring service to ensure patient records were accurately captured in the CRFs. All complications were reviewed and adjudicated by an independent clinical events committee (CEC) consisting of physicians experienced with TLE procedures.

### Lead extraction procedure

Participating centers used a consistent approach to TLE procedures. TLE was performed under general anesthesia. A superior approach via the implant-related vein was the first-line method with most targeted leads. The skin incision was chosen to achieve appropriate coaxial alignment of the extraction sheath with the targeted lead(s) in the subsequent lead extraction procedure. The leads were dissected free from adhesions inside the generator pocket and the fixation sutures were removed.

**Figure 1:**
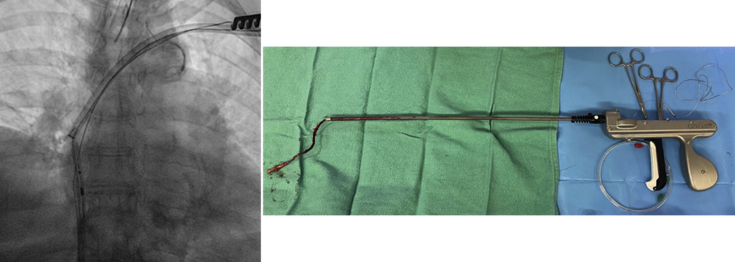
Bidirectional rotational transvenous lead extraction device used to extract long indwelling leads.

The appropriate mechanical TLE devices were chosen per physician discretion. First, simple traction (as defined in the 2018 European Heart Rhythm Association expert consensus statement^1^), was attempted in all cases and served as a diagnostic measure to determine whether adhesions were present. If simple traction was unsuccessful, a locking stylet (Liberator® Beacon® Tip Locking Stylet, Cook Medical, Bloomington, IN) was introduced into the inner lumen of the lead and deployed. To provide greater lead control, a compression coil (One-Tie® Compression Coil, Cook Medical) or a Bulldog™ Lead Extender (Cook Medical) could be used. Fibrous adhesions surrounding the lead were dissected using the Evolution Mechanical Dilator Sheath Set or Evolution RL Controlled-Rotation Dilator Sheath Set, to facilitate lead extraction (Cook Medical, Figure 1).

In cases of extensive scarring or calcification at the vessel entry site, the Evolution Shortie Mechanical Dilator Sheath Set or Evolution Shortie RL Controlled-Rotation Dilator Sheath Set was used. When a superior approach was not possible (or failed), a femoral approach using the Needle’s Eye Snare® (Cook Medical) was used to remove leads or lead fragments. Some leads were removed using a combination of superior and femoral extraction approaches. Lastly, lead extraction time was captured and defined as the time elapsed from the point when a locking stylet touched the targeted lead until the lead was removed from the vascular space or the procedure was terminated. Efficiency of lead extraction was evaluated by comparing the lead indwell time with the extraction time per lead or per TLE procedure.

### Objectives, outcomes, and definitions

The study objectives were to assess the rates of clinical success and complete procedural success as well as to evaluate procedural efficiency and complications through study follow-up.

Success measures and complications were defined in accordance with current Heart Rhythm Society and European Heart Rhythm Association recommendations.^1^^,^^2^ Specifically, complete procedural success was defined as the removal of the targeted lead and all lead material from the vascular space, with the absence of permanently disabling complications or procedure-related death.^1^^,^^2^ Clinical success was defined as the removal of all targeted leads and lead material from the vascular space, or retention of a small portion of the lead (fragment ≤4 cm) that did not impact the outcome goals of the procedure.^1^^,^^2^

Major complications were defined as any outcomes related to the procedure that were life threatening or resulted in death (cardiac or noncardiac).^1^ Major complications also included unexpected events that caused persistent or significant disability, events that required inpatient hospitalization or prolongation of existing hospitalization, or any event that required surgical intervention to prevent any of the outcomes listed above.^1^ Minor complications included any undesired event related to the procedure that required medical intervention or minor procedural intervention that did not limit persistently or significantly alter the patient’s function or threaten the life of the patient.^1^

An independent CEC adjudicated all reported complications to assess major complications and determined the relationship of the event to the lead extraction procedure, device(s), or patient’s pre-existing conditions. Both the CEC and clinical monitoring were managed by a clinical research organization that is independent of Cook Medical.

### Data analysis

Continuous variables were summarized with mean ± standard deviation or median with interquartile range (IQR), with *P* values calculated using a Median test. Categorical variables were reported as counts and percentages. As appropriate, the number of observations represented the number of patients or the number of leads. All analyses were performed using SAS version 9.4 (SAS Institute Inc, Cary, NC). A *P* value < .05 was considered significant. The data analyses were outlined by the study investigators and Cook Research Incorporated. Statistical analysis was performed at Cook Research Incorporated, and study investigators provided input and review of all study data. Study investigators had full access to all study data and approved all results provided in this manuscript.

## Results

A total of 230 patients (67.4% male, mean age 64.3 ± 14.4 years) underwent mechanical TLE from October 2018 to January 2020 at 10 medical centers in the United States and Europe (Table 1). All patients underwent a follow-up visit approximately 4 weeks post-procedure, as determined per standard of care (mean 31.2 ± 19.5 days; median 29 days, range 6–150 days). Complete procedural data were collected for all 230 patients. Follow-up data were collected for 228 patients (99.1%); 1 patient was lost to follow-up and 1 withdrew from the study following the TLE procedure.

**Table 1:** Patient demographics and comorbid conditions

Patient characteristics	Mean ± SD (n, min–max)
Age (years)	64.3 ± 14.4 (230, 22–92)
BMI†	29.6 ± 6.7 (227, 16.5–59.1)

	Percent of patients (n/N)
Male	67.4% (155/230)
Ejection fraction under 35%	32.2% (74/230)
Coronary artery disease	52.6% (121/230)
Cardiomyopathy	56.1% (129/230)
Congestive heart failure	65.7% (151/230)
NYHA classification	
1	17.2% (26/151)
2	42.4% (64/151)
3	37.1% (56/151)
4	3.3% (5/151)
Diabetes mellitus	29.6% (68/230)
Hypertension	67.4% (155/230)
Renal failure/insufficiency	28.3% (65/230)
Pacing dependency (HR under 40 beats/min)	20.0% (46/230)

BMI = body mass index; HR = heart rate.

† BMI information was unavailable for 3 patients.

**Figure 2:**
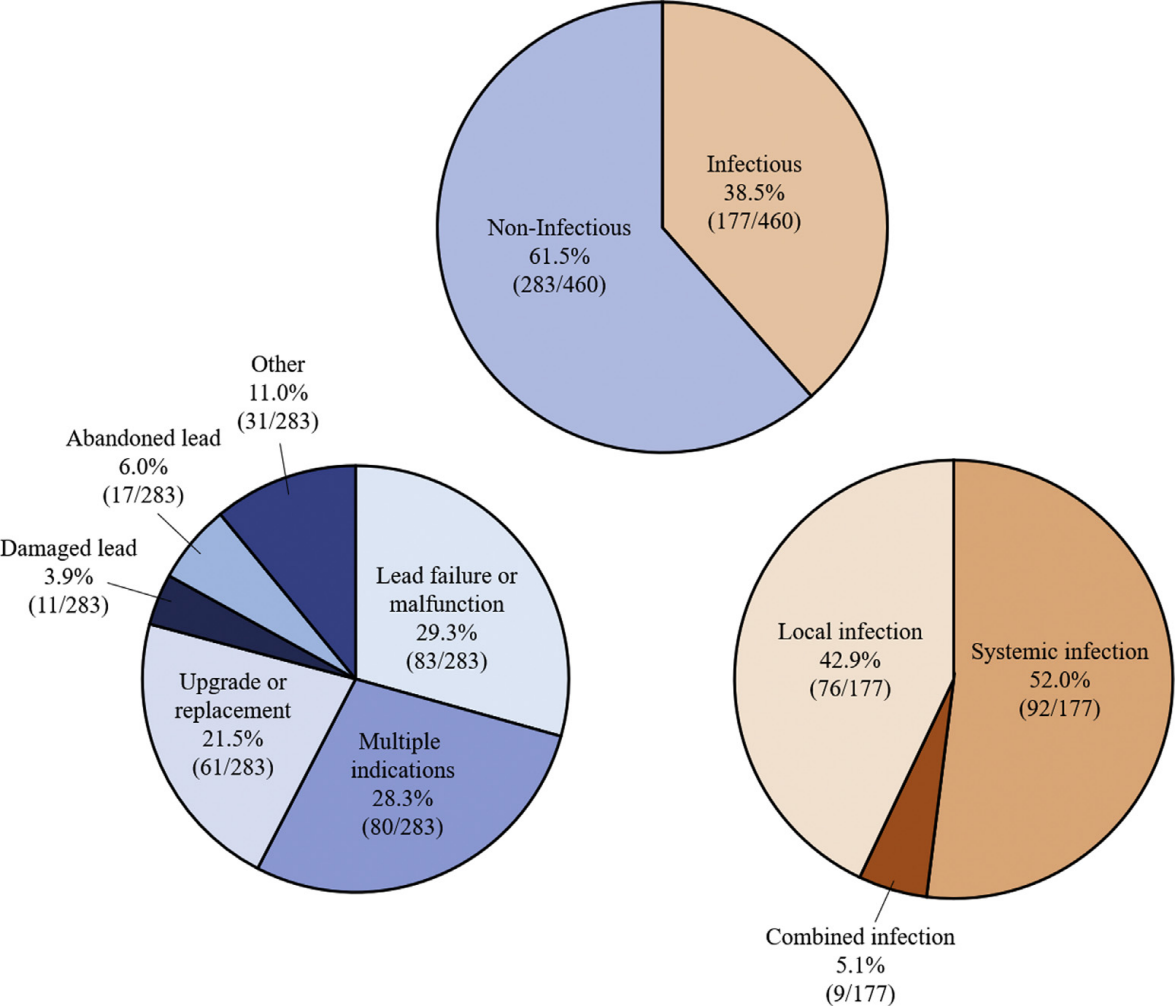
Indications for lead extraction. Multiple indications could be reported per lead.

In all, 460 leads were extracted with a median dwell time of 7.4 years (IQR 4–12 years, range 0.1–35.6 years). Lead characteristics and indications for lead extraction are available in Table 2 and Figure 2, respectively. Systemic infection was the single most common indication for lead removal (Figure 2). The majority of TLE procedures were scheduled for noninfectious indications, with leads removed most often for nonemergent indications, including lead failure/malfunction or need for device upgrade (Figure 2).

**Table 2:** Lead and device characteristics

Lead characteristics	Mean ± SD (n, range), Median [IQR]
Implant duration (years)†	
Mean	8.8 ± 6.0 (453, 0.1–35.6)
Median	7.4 [IQR 4–12]
Leads extracted per patient	
Mean	2.0 ± 0.9 (230, 1–6)
Median	2 [IQR 1–3]
Lead extraction time per lead (minutes)	
Mean	12.4 ± 22.1 (458, 1–187)
Median	4 [IQR 1–13]
Lead extraction time per procedure (minutes)	
Mean	18.0 ± 24.5 (230, 1–189)
Median	10 [IQR 4–22]

	Percent of leads (n/N)
Right atrial leads	34.6% (159/460)
Right ventricular leads	55.7% (256/460)
Coronary sinus leads	9.3% (43/460)
Superior vena cava	0.4% (2/460)
ICD leads	33.7% (155/460)
Dual-coil ICD leads	55.5% (86/155)
Single-coil ICD leads	44.5% (69/155)
Lead fixation	
Active	81.5% (375/460)
Passive	18.5% (85/460)

ICD = implantable cardioverter-defibrillator; IQR = interquartile range.

† Unavailable implant date for 7 leads and unavailable extraction time for 2 leads.

A superior approach was used in 90.9% (418/460) of lead extractions with a multistep method (Table 3). Simple traction was sufficient for removing 4.5% (19/418) of leads. For leads that could not be removed using simple traction, a locking stylet and compression coil were used to extract 7.4% (31/418) of leads. Most leads were extracted using a bidirectional rotational lead extraction sheath (88.0%, 368/418). An isolated femoral approach using the Needle’s Eye Snare was required to extract 1.5% (7/460) of leads, including leads that were not accessible using the superior approach (1.1%, 5/460) or were fragmented or damaged during the TLE procedure (0.4%, 2/460). A small minority of leads (7.6%, 35/460) were extracted using a combination of approaches, either sequentially or in a “hybrid approach” using the superior and femoral approaches simultaneously.

**Table 3:** Approaches used during lead extraction

Lead extraction approach	Percentage of leads (n/N)
Superior approach alone	90.9% (418/460)
Simple traction	4.5% (19/418)
Locking stylet only	7.4% (31/418)
Bidirectional rotational lead extraction	88.0% (368/418)
Isolated femoral approach	1.5% (7/460)
Combined approaches	7.6% (35/460)

Complete procedural success was achieved for 96.3% (443/460) of leads. Fourteen leads were incompletely extracted, and 3 leads were completely removed but the patient experienced a procedure-related death 2 days post-procedure. Partial lead extraction (lead with <4 cm remnant) was achieved in 2.4% (11/460) of leads (Table 4). Retention of ≥4 cm of lead material occurred in 0.7% (3/460) of leads; this occurred in patients with complicating factors such as a history of coronary artery disease, congestive heart failure, or right ventricle implantable cardioverter-defibrillator leads with lead indwell times of ≥83 months. The ≥4 cm lead fragments did not result in any undesired outcomes or lead extraction–related complications. Overall, clinical success was achieved in 98.7% (227/230) of procedures and complete procedural success was achieved in 93.9% (216/230) of patients.

**Table 4:** Clinical and procedural success

Clinical and procedural success	Percentage of patients or leads (n/N)
Clinical success, per patient	98.7% (227/230)
Complete procedural success, per lead	96.3% (443/460)
Partial lead removal (< 4 cm remnant)	2.4% (11/460)

### Extraction time

**Figure 3:**
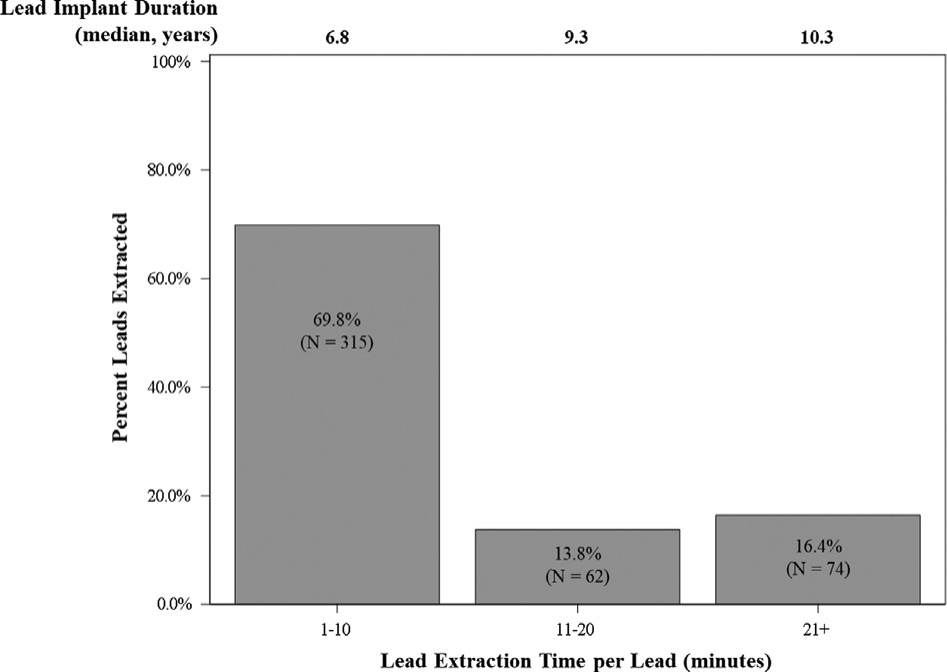
Lead extraction time vs lead implant duration. (Unavailable lead extraction time or lead implant duration for 9 leads.)

The ease of extraction when using mechanical TLE devices was assessed by comparing lead indwell time with lead extraction time. Figure 3 provides information on lead extraction time per lead as it relates to lead implant duration. Lead indwell time and extraction time were reported for 98.0% of leads (451/460). The median lead indwell time was 7.4 years (IQR: 4–12 years, range 0.1–35.6 years). The median extraction time per procedure was 10 minutes (IQR: 4–22 minutes, range 1–189 minutes) and median extraction time per lead was 4 minutes (IQR: 1–13 minutes, range 1–187 minutes). Most leads were extracted within 10 minutes, and these leads had a median lead implant duration of 6.8 years (69.8%, 315/451) (Figure 3). Leads that took 11 minutes or longer to extract had a median implant duration of 9.3 years or longer (30.2%, 136/451) (Figure 3). The median indwell time for leads removed within 10 minutes was significantly shorter than leads with extraction time of 11 minutes or longer (*P* < .0001). Overall, 83.6% (377/451) of leads were extracted within 20 minutes.

### Complications

Of the 55 complications reviewed by the CEC, 13 patients (5.7%, 13/230) experienced major complications either during procedure or follow-up that were CEC-adjudicated as related to the lead extraction procedure or to the device(s) (Supplemental Table 1). Six patients experienced complications that were adjudicated as device-related (2.6%, 6/230) (Table 5). Complications per patient included pericardial effusion, hemothorax requiring a chest tube and blood transfusion, a severed introducer sheath that led to a foreign body snare removal procedure, 2 cardiac injury events, and a tricuspid valve flail. One patient experienced a cardiac injury at the superior vena cava (SVC) /right atrial junction and another patient had an injury to the atrial septum during the TLE procedure. The tricuspid valve flail event on day 6 post-procedure was the only device-related major complication reported during follow-up. The 3 patients who experienced cardiac injuries and tricuspid valve flail (1.3%, 3/230) required open surgical repair. The remaining 3 patients (1.3%, 3/230) did not require open surgical repair and instead were treated with pericardiocentesis, chest tube placement, and endovascular snaring of a foreign body. No patients experienced isolated extrapericardial SVC injuries. Further details regarding CEC-adjudicated major complications related to lead extraction are displayed in Supplemental Table 1.

**Table 5:** Clinical events committee–adjudicated device-related major complications

Device-related major complications	Percentage of patients (# of events)	
	Procedural events	Events during follow-up
Complications that required open heart surgery		
Cardiac injury of the interatrial septum	0.4% (1)	0.0% (0)
Cardiac injury at the SVC/RA junction	0.4% (1)	0.0% (0)
Tricuspid valve flail	0.0% (0)	0.4% (1)
Total	0.9% (2)	0.4% (1)
Complications that did not require open heart surgery		
Pericardial effusion requiring pericardiocentesis	0.4% (1)	0.0% (0)
Hemothorax requiring placement of a drain	0.4% (1)	0.0% (0)
Introducer sheath severed by Cook Evolution RL (Cook Medical, Bloomington, IN) (removed by snare)	0.4% (1)	0.0% (0)
Total	1.3% (3)	0.0% (0)

Rates represent complications in 230 enrolled patients.

### Mortality

No patient deaths occurred during the study procedure. Eight (3.5%, 8/230) patient deaths were reported within follow-up spanning 2-44 days post-procedure. Seven deaths (3.0%, 7/230) were CEC-adjudicated as not related to lead extraction, but instead related to pre-existing conditions such as infection or comorbid conditions. Moreover, 7 patients who died post-procedure had leads removed owing to systemic infection, and 3 of these patients died of sepsis or septic shock. One patient with systemic infection died 2 days post-procedure and the death was CEC-adjudicated as related to the lead extraction procedure. The patient was a 76-year-old woman with a history of coronary artery disease, congestive heart failure, chronic obstructive pulmonary disease, and long-term steroid use. This patient had 3 leads extracted for systemic infection including cardiovascular implantable electronic device–related endocarditis. During the procedure, the patient developed severe bleeding at a femoral arterial access site that was not utilized to extract the cardiac leads. All 3 leads were completely extracted using the superior approach via the subclavian vein. The severe bleeding at the femoral access site led to cardiogenic shock and the patient died 2 days post-procedure. Overall, no patient deaths were CEC-adjudicated as related to lead extraction device use.

## Discussion

Previous retrospective studies reported favorable safety and efficacy results for patients undergoing mechanical TLE, including using rotational TLE devices^6^^,^^8^^,^^9^^,^^13^^,^^14^^,^^16^; however, prospective TLE studies have been scarce. The aim of the RELEASE study was to perform a prospective, international, multicenter, observational clinical investigation on real-world patients to evaluate the safety and effectiveness of mechanical TLE, including the use of rotational TLE devices. The study design incorporated additional measures to more accurately collect and assess patient data, including independent clinical monitoring and source verification of patient data and adjudicating all reported complications by an independent CEC. This rigorous study provides real-world outcome data for mechanical TLE.

Patients enrolled in the RELEASE study had similar demographics and medical history with respect to other TLE studies.^4^^,^^6^^,^^8^^,^^14^^,^^15^ The age (mean age, 64.3 years) and preponderance of male patients with a high prevalence of congestive heart failure (65.7%), diabetes mellitus (29.6%), and coronary artery disease (52.6%) enrolled in the RELEASE study were similar to other TLE studies.^4^^,^^6^^,^^8^^,^^14^^,^^15^ Lead characteristics (pacemaker vs implantable cardioverter-defibrillator leads and fixation type) were also similar to other studies, with the exception of a longer mean implant duration of 8.8 ± 6.0 years.^6^^,^^8^^,^^14^^,^^15^ The most common TLE technique used in the RELEASE study was the superior approach with a bidirectional rotational TLE sheath. The bidirectional rotational TLE procedures represented in the RELEASE study were complex in a high-risk population. Historically, infection was the predominant indication for undergoing TLE procedures whereas the majority of patients in the RELEASE study had leads extracted for noninfectious indications.^4^^,^^6^^,^^15^ Witte and colleagues^10^ observed an increase in lead malfunction as an indication for lead extraction and suggested a potential trend toward lead extraction for noninfectious indications. This is supported by an increase in lead removal for noninfectious indications in several multicenter studies over the course of the past decade. Infectious indications were reported in 56.9% of the leads extracted in the 2010 LExICon study.^4^ The authors of the ELECTRa study^15^ (2017) and a large, multicenter Italian registry^8^ (2018) reported lead extractions for infectious indications in 52.8% and 50.8% of lead extractions, respectively. Finally, the authors of the 2020 PROMET study^6^ reported that a total of 46.0% of leads were extracted for infectious indications. Therefore, the RELEASE study data represent a growing trend in lead management practice, where leads are increasingly being extracted for noninfectious indications.^4^^,^^6^^,^^15^

The RELEASE study results revealed high success rates (clinical success rate per patient, 98.7%; complete procedural success per lead, 96.3%) despite the long indwell time of targeted leads. Compared with similar TLE studies, this study includes patients with longer lead indwell times and reports a similar success rates.^4^^,^^15^^,^^17^

Mechanical rotational TLE is often perceived to require longer extraction times owing to presumed slower action of disruption of fibrotic adhesions. The authors of the ELECTRa study reported a median extraction time of 19.0 minutes (IQR 6.0–40.0 minutes) with a median implant duration of 5 years using various TLE techniques, including manual traction, locking stylets alone, and laser or mechanical sheaths.^15^ The median extraction time for the RELEASE study was 10 minutes per TLE procedure (IQR 4–22 minutes) and 4 minutes per lead (IQR 1–13 minutes). Nearly 70% of the leads were extracted within 10 minutes, despite a median lead implant duration of 7.4 years (Figure 3 and Table 2). Furthermore, leads indwelling longer than 7.4 years (228 leads) had a median extraction time of 7 minutes, indicating that mechanical TLE, especially rotational TLE, is an efficient technique for lead extraction.

TLE is frequently perceived as a high-risk procedure.^1^^,^^2^ Accordingly, it is recommended that TLE be performed in specialized centers with well-trained teams and surgical back-up. The RELEASE study recorded all complications that occurred procedurally and during follow-up (around 30 days post-procedure), reflecting events that are not typically reported in TLE studies. Overall, 13 patients (5.7%, 13/230) experienced major complications that were CEC-adjudicated as related to the lead extraction procedure or to the device(s). Seven patients (3.0%, 7/230) experienced major complications during the lead extraction procedure and 6 patients (2.6%, 6/230) experienced major complications following the day of the procedure (Supplemental Table 1). The rate of procedure-related major complications occurring within 1 day of the procedure was 0.9% (2/230), which is in line with previous studies.^4^^,^^6^^,^^13^, ^14^, ^15^ Additionally, procedure-related major complications observed during follow-up included 2.2% (5/230) of patients (Supplemental Table 1). Device-related major complications were reported in 2.6% (6/230) of patients, half of whom (1.3%, 3/230) required a cardiac surgical procedure requiring sternotomy or thoracotomy, including a tricuspid valve operation 6 days after the initial lead extraction (Table 5). The rate of major complications related to lead extraction observed procedurally is slightly higher compared to other TLE studies.^4^^,^^6^^,^^8^^,^^13^^,^^15^ The RELEASE study may have captured more complications owing to the controlled study design (prospective data collection, source data verification by an independent clinical monitoring service, and adjudication of all documented events by an independent CEC). The RELEASE study design could serve as a benchmark for future lead extraction studies to better understand the safety of using lead extraction devices.

Extrapericardial SVC injuries have been identified as the most lethal complication encountered in TLE procedures.^18^ A special occlusion balloon has been introduced to improve outcomes after this complication.^18^ In 1 study reporting on the efficacy of the occlusion balloon that included 116 surgically confirmed SVC events, 90.5% of the SVC injuries occurred in patients who underwent TLE procedures involving a laser extraction sheath.^19^ In this context, it is important to note that no isolated SVC injury occurred in the RELEASE study. These data are in line with the results of a MAUDE database analysis^11^ and the PROMET study^6^ suggesting that there is a lower risk of isolated SVC injury with use of rotational TLE tools compared with the use of laser sheaths.

No procedural mortality occurred during the RELEASE study. Although 8 patient deaths were reported during follow-up, 7 deaths were CEC-adjudicated as not related to lead extraction and were caused by pre-existing conditions such as infection or comorbid conditions. One patient death was CEC-adjudicated as related to the lead extraction procedure; however, the mechanical TLE devices were not involved in the complications that led to death (Supplemental Table 1). Overall, the complications and mortality observed in the RELEASE study demonstrate the low risk of mortality and support the safety of using mechanical TLE devices, including bidirectional rotational TLE tools. The highly controlled RELEASE study design encourages that future TLE studies prospectively collect follow-up safety data to better understand complications following the TLE procedure.

There are some limitations of the RELEASE study. First, this was an observational study to research the performance and outcomes of mechanical TLE devices; therefore, there was no comparator group. Patient eligibility was based on an investigator’s intention to treat a patient with Cook lead extraction device(s) and does not represent consecutive patient enrollment. Therefore, the patient cohort may not be representative of all patients requiring transvenous lead extraction and who might be treated by other methods. The RELEASE study only included centers within Europe and the United States and representing TLE procedure in 2 regions; therefore, this study does not provide worldwide data on patients undergoing TLE. Moreover, the sample size of the RELEASE study was limited, and larger studies with additional low- and high-volume centers are warranted.

## Conclusion

The RELEASE clinical study demonstrates that use of mechanical TLE devices, especially use of bidirectional rotational lead extraction sheaths, are effective, efficient, and safe tools for TLE. This study represents an advance in TLE trial design, as this study emphasizes the need for comprehensive follow-up and adjudication of all complications by an independent CEC with experience in TLE procedures. Prospective, multicenter, monitored clinical studies will provide a more accurate understanding of the effectiveness and safety of the use of TLE devices.

## Acknowledgments

The authors thank all patients who participated in this study, as well as all the study investigators and research personnel. The authors thank the following individuals from Cook Research Incorporated for their contributions to the development of this manuscript: Nicholas Dey and John Weidle for providing critical review of the manuscript.

## Funding Sources

This study was sponsored by 10.13039/100010479Cook Medical.

## Disclosures

Dr Sharma declares payment to institution for speaker fees, advisory board, consultancy, research for Cook Medical, Abbot Medical, and Boston Scientific. Dr Lee declares research funding from Boston Scientific, Cook Medical, Medtronic, and Zoll Medical and honoraria from Biotronik, and Zoll Medical. Dr Garg has received honorarium from Cook Medical. Dr Peyton is consultant for Cook Medical. Dr Schuler discloses honoraria from Cook Medical as member of advisory board, speaker, research and development consultant, and physician proctor. Dr Mason declares payment related to her activity as consultancy, advisory board fees, and investigator for Boston Scientific, Cook Medical, and Medtronic. Dr Delnoy has been paid a consulting fee by Cook, Medtronic, Boston Scientific, Biotronik, Microport, and Abbott and is on their speakers’ bureau and received funding for a research assistant. Dr Gallagher has received research funding from Medtronic Inc and Attune Medical and has acted as a consultant or paid speaker for Medtronic, Cook Medical, Biosense Webster, Biotronik, and Attune Medical. Dr Hariharan declares research funding from Medtronic, Boston Scientific, Abbott, Biotronki, and Cook Medical. Dr Schaerf is consultant and instructor for Cook Medical. R. Du and Dr Serratore are salaried employees of Cook Research Incorporated, a Cook Group Company. Dr Starck declares payment to his institution related to his activity as speaker fees, honoraria, consultancy, advisory board fees, investigator, committee member from Abiomed, Angiodynamics, Medtronic, Philips, Biotronik, Liva Nova, and Cook Medical and departmental or institutional research funding from Cook Medical.

## Authorship

All authors attest they meet the current ICMJE criteria for authorship.

## Patient Consent

All patients provided written informed consent prior to enrollment.

## Ethics Statement

This study was conducted in accordance with the Declaration of Helsinki, ISO 14155, and applicable local regulations. All local institutional review boards (US) / ethics committees (EU) approved the study protocol.
