# Supplementary material for: Performance and outcomes of transvenous rotational lead extraction: Results from a prospective, monitored, international clinical study
Source: Heart Rhythm O2. 2021 Mar 2;2(2):113–21. doi: 10.1016/j.hroo.2021.02.005 (PMC8183877; doi:10.1016/j.hroo.2021.02.005)
Supplement: Supplemental Table 1 [file mmc1.docx]

**Supplemental Table 1. Major Complication related to Lead Extraction**

| **Patient Demographics** | **Device and**  **Lead Information** | **Indication for Extraction** | **Indwell Time of Oldest Lead (months)** | **Devices Used** | **Days to Complication(s)** | **Complication and Treatment** | **Outcomes** | **CEC Adjudication** |
| --- | --- | --- | --- | --- | --- | --- | --- | --- |
| 81 years, male | Single chamber pacemaker, 3 leads | Lead failure/ malfunction; upgrade/  replacement two leads adhered to target lead | 49 | Locking stylet, compression coil, lead extender, bidirectional rotational sheath | 0 | Vascular avulsion or tear (subclavian vein tear); Surgical repair | Complication resolved; Patient died 15 days post-procedure | Complication related to lead extraction procedure; Death not related to lead extraction |
| 45 years, female | Single chamber pacemaker, 2 leads | Need for MRI compatible device | 83 | Locking stylet, compression coil, bidirectional rotational sheath, femoral snare | 0 | Pericardial effusion; Pericardiocentesis | Resolved without sequelae | Related to lead extraction device(s) use |
| 50 years, female | CRT-D, 2 leads | Lead failure/  malfunction | 153 | Locking stylet, compression coil, bidirectional rotational sheath | 0 | Cardiac injury at SVC/RA junction; Pericardiocentesis and thoracotomy | Resolved without sequelae | Related to lead extraction procedure and device(s) use |
| 74 years, male | Single chamber ICD, 2 leads | Lead failure/  malfunction | 250 | Locking stylet, compression coil, bidirectional rotational sheath, stabilization sheath | 0 | Introducer sheath severed by  Cook Evolution RL;  Foreign body removed by snare | Resolved without sequelae | Related to lead extraction device(s) use |
| 92 years, male | Single chamber pacemaker, 1 lead | Infection, local | 190 | Locking stylet, compression coil, mechanical sheath, bidirectional rotational sheath, stabilization sheath | 0 | Bleeding event following heparin administration for implant of leadless device; Vein repaired with sutures and electrocautery | Resolved without sequelae | Related to lead extraction procedure |
| 53 years, female | Single chamber pacemaker, 3 leads | Infection, local | 380 | Locking stylet, compression coil, lead extender, bidirectional rotational sheath, stabilization sheath, femoral snare | 0 | Atrial septum tear, cardiac arrest, and embolus to left main coronary artery; vascular injury at attempted bypass cannulation site; Cardiopulmonary bypass, ECMO, atrial septal defect repair, left heart catheter and right femoral artery repair | Resolved without sequelae | Related to lead extraction procedure and device(s) use |
| 76 years, female | Dual chamber pacemaker, 3 leads | Infection, systemic | 39 | Locking stylet, compression coil, bidirectional rotational sheath | 0 | Bleeding from femoral arterial access site, cardiogenic shock; Transfusion and high dose of vasopressors, bicarb, intubation/sedation, stress dose steroids | Bleeding resolved; Patient died 2 days post-procedure | Complications and death related to lead extraction procedure |
| 71 years, female | Dual chamber ICD, 2 leads | Lead failure/  malfunction; damaged lead | 169 | Locking stylet, compression coil, bidirectional rotational sheath | 1 | Hemothorax, RLE DVT, and pulmonary embolus; Chest tube and IVC filter placement | Resolved without sequelae | Related to lead extraction procedure and device(s) use |
| 80 years, male | Dual chamber pacemaker, 3 leads | Infection, local | 423 | Locking stylet, lead extender, compression coil, bidirectional rotational sheath | 2 | Bleeding at pocket site following pacemaker implant; Left chest wound exploration, wound packed with hemostatic material and transfusion | Resolved without sequelae | Related to lead extraction procedure |
| 48 years, female | Single chamber ICD, 2 leads | Infection, systemic | 73 | Locking stylet, compression coil, mechanical sheath, bidirectional rotational sheath, stabilization sheath | 2 | Venous thrombosis and  pulmonary embolism;  Heparin and transitioned to rivaroxaban | Complication resolved; Patient died 29 days post-procedure | Complication related to lead extraction procedure; Death not related to lead extraction |
| 69 years, male | Single chamber pacemaker, 2 leads | Infection, systemic | 82 | Locking stylet, compression coil, bidirectional rotational sheath, stabilization sheath | 2 | Large blood clot surgically  removed from pocket site;  Surgical pocket washout | Resolved without sequelae | Related to lead extraction procedure |
| 65 years, female | Dual chamber ICD, 2 leads | Lead failure/  malfunction | 92 | Locking stylet, compression coil, bidirectional rotational sheath, stabilization sheath | 6 | Tricuspid valve flail;  Surgical repair of tricuspid valve | Resolved without sequelae | Related to lead extraction device(s) use |
| 76 years, female | Single chamber pacemaker, 2 leads | Tricuspid regurgitation | 68 | Locking stylet, compression coil, bidirectional rotational sheath | 35 | Former pocket site infection;  Vacuum Assisted Closure therapy  and antibiotics | Resolved without sequelae | Related to lead extraction procedure |
